# Ethyl 7-chloro­methyl-5-(2-chloro­phen­yl)-7-hydr­oxy-2-methyl­sulfanyl-4,5,6,7-tetra­hydro-1,2,4-triazolo[1,5-*a*]pyrimidine-6-carboxyl­ate

**DOI:** 10.1107/S1600536809039373

**Published:** 2009-10-10

**Authors:** Shao-wei Huang

**Affiliations:** a Key Laboratory of Pesticides and Chemical Biology of the Ministry of Education, College of Chemistry, Central China Normal University, Wuhan 430079, People’s Republic of China

## Abstract

In the title compound, C_16_H_18_Cl_2_N_4_O_3_S, the five-membered ring is almost planar [maximum deviation = 0.011 (3) Å] and the six-membered ring adopts an envelope conformation. In the crystal structure, N—H⋯N, O—H⋯N and C—H⋯O inter­actions link mol­ecules into a three-dimensional network.

## Related literature

For general background to tetra­hydro triazolo[1,5-*a*]pyrimi­dine derivatives as potential biologically active compounds, see: Pryadeina *et al.* (2004 ▶). For related structures, see: Chen *et al.* (2005 ▶); Hu *et al.* (2005 ▶). For bond-length data, see: Allen *et al.* (1987 ▶).
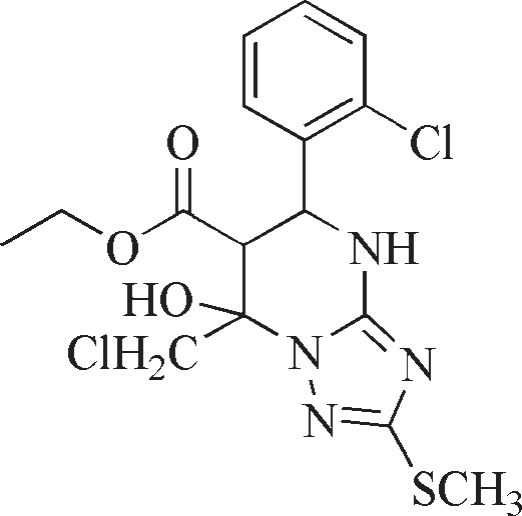


## Experimental

### 

#### Crystal data

C_16_H_18_Cl_2_N_4_O_3_S
*M* *_r_* = 417.30
Triclinic, 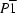

*a* = 8.4534 (14) Å
*b* = 10.5082 (17) Å
*c* = 12.0846 (19) Å
α = 66.660 (3)°
β = 79.519 (3)°
γ = 84.795 (3)°
*V* = 969.0 (3) Å^3^
*Z* = 2
Mo *K*α radiation
μ = 0.47 mm^−1^
*T* = 292 K
0.30 × 0.20 × 0.10 mm

#### Data collection

Bruker SMART 4K CCD diffractometer
Absorption correction: none
6772 measured reflections
3759 independent reflections
2661 reflections with *I* > 2σ(*I*)
*R*_int_ = 0.025

#### Refinement

*R*[*F* ^2^ > 2σ(*F* ^2^)] = 0.053
*wR*(*F* ^2^) = 0.141
*S* = 1.08
3759 reflections
249 parameters
H atoms treated by a mixture of independent and constrained refinement
Δρ_max_ = 0.43 e Å^−3^
Δρ_min_ = −0.33 e Å^−3^

### 

Data collection: *SMART* (Bruker, 2001 ▶); cell refinement: *SAINT* (Bruker, 2001 ▶); data reduction: *SAINT*; program(s) used to solve structure: *SHELXS97* (Sheldrick, 2008 ▶); program(s) used to refine structure: *SHELXL97* (Sheldrick, 2008 ▶); molecular graphics: *PLATON* (Spek, 2009 ▶); software used to prepare material for publication: *SHELXTL* (Sheldrick, 2008 ▶).

## Supplementary Material

Crystal structure: contains datablocks I, global. DOI: 10.1107/S1600536809039373/hb5113sup1.cif

Structure factors: contains datablocks I. DOI: 10.1107/S1600536809039373/hb5113Isup2.hkl

Additional supplementary materials:  crystallographic information; 3D view; checkCIF report

## Figures and Tables

**Table 1:** Hydrogen-bond geometry (Å, °)

*D*—H⋯*A*	*D*—H	H⋯*A*	*D*⋯*A*	*D*—H⋯*A*
N1—H1⋯N4^i^	0.93 (3)	2.04 (3)	2.969 (3)	172 (2)
O3—H3*A*⋯N3^ii^	0.77 (3)	2.05 (3)	2.806 (3)	170 (3)
C16—H16*A*⋯O3^ii^	0.96	2.47	3.290 (4)	143

Symmetry codes: (i) 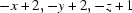; (ii) 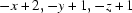.

**Fig. 1.**
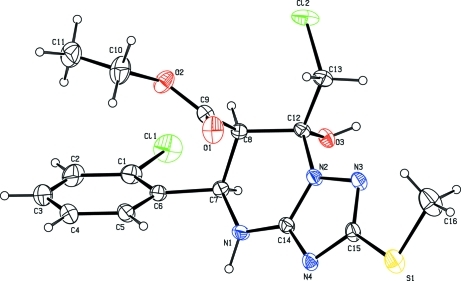
The molecular structure of (I) with displacement ellipsoids drawn at the 50% probability level.

**Fig. 2.**
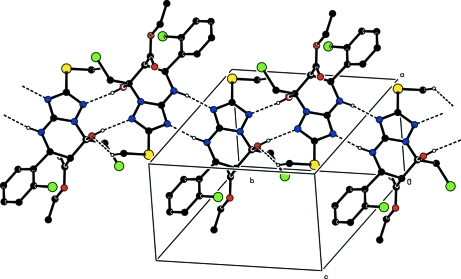
A partial packing diagram of (I). Hydrogen bonds are shown as dashed lines. H atoms not involved in hydrogen bonding have been omitted for clarity.

## supplementary crystallographic information

### Comment

In recent years, tetrahydro triazolo1,5-a]pyrimidine derivatives nave attracted interest as potential biologically active compounds (Pryadeina *et al.*, 2004). In this paper, we present the structure of one such analogue, the title compound, (I) (Fig. 1), in which the bond lengths (Allen *et al.*, 1987) and angles are within normal ranges.

The bicyclic triazolopyrimidine system is not on the same plane because they can not form a conjugated system. Ring A (N2-4/C14-15) is close to planarity with a maximum deviation of 0.011Å for C15. Rng B (N1-2/C7-8/C12/C14) dopt envelope conformations with atom C8 displaced by 0.333 (3) Å from the plane of the other ring atoms. The dihedral angle between the Ring (C1-C6) and the ring A is 51.86°.

In the crystal structure, weak N-H···N, O-H···N and C-H···O interactions link the molecules into a three-dimensional network (Fig. 2).

### Experimental

A solution of 4-chloro acetylacetic ester (1 mmol), 2-chloro benzaldehyde (1 mmol), and 3-amino-5-methylthio-1,2,4-triazole (1mmol) in water (3 ml) containing a catalytic amount of TSA was heated under 353 K for 10 h. The resulting mixture was extracted with CH_2_Cl_2_ and the extra was dried over sodium sulfate, filtered, the filtrate was condensed under reduced pressure and the residue was purified by chromatography on SiO_2_ to afford the title compound. Colourless blocks of (I) were grown from an acetone solution at 293K. ^1^H NMR (CDCl_3_, 400 MHz): σ 7.03-7.40(m, 4 H), 6.42(s, 1H), 4.99(d, 1H), 4.35(d, 1H), 4.00(q,2 H), 3.91(d, 1H), 3.05(d, 1H), 2.35(s, 3H), 1.03(t,3H).

### Refinement

The N- and O-bound H atoms were located in a difference map and freely refined with fixed isotropic displacement parameters. All other H atoms were positioned geometrically, with C-H = 0.93, 0.97 and 0.96 Å for aromatic, methylene and methyl H, respectively, and constrained to ride on their parent atoms, with U_iso_(H) = 1.2U_eq_(C) or 1.5U_eq_(methyl C).

### Crystal data

**Table:**

C_16_H_18_Cl_2_N_4_O_3_S	*Z* = 2
*M**_r_* = 417.30	*F*(000) = 432
Triclinic, *P*1	*D*_x_ = 1.430 Mg m^−^^3^
Hall symbol: -P 1	Mo *K*α radiation, λ = 0.71073 Å
*a* = 8.4534 (14) Å	Cell parameters from 2018 reflections
*b* = 10.5082 (17) Å	θ = 2.5–24.1°
*c* = 12.0846 (19) Å	µ = 0.47 mm^−^^1^
α = 66.660 (3)°	*T* = 292 K
β = 79.519 (3)°	Block, colourless
γ = 84.795 (3)°	0.30 × 0.20 × 0.10 mm
*V* = 969.0 (3) Å^3^	

### Data collection

**Table:**

Bruker SMART 4K CCD diffractometer	2661 reflections with *I* > 2σ(*I*)
Radiation source: fine-focus sealed tube	*R*_int_ = 0.025
graphite	θ_max_ = 26.0°, θ_min_ = 1.9°
φ and ω scans	*h* = −10→10
6772 measured reflections	*k* = −12→12
3759 independent reflections	*l* = −14→14

### Refinement

**Table:**

Refinement on *F*^2^	Primary atom site location: structure-invariant direct methods
Least-squares matrix: full	Secondary atom site location: difference Fourier map
*R*[*F*^2^ > 2σ(*F*^2^)] = 0.053	Hydrogen site location: inferred from neighbouring sites
*wR*(*F*^2^) = 0.141	H atoms treated by a mixture of independent and constrained refinement
*S* = 1.08	*w* = 1/[σ^2^(*F*_o_^2^) + (0.07*P*)^2^] where *P* = (*F*_o_^2^ + 2*F*_c_^2^)/3
3759 reflections	(Δ/σ)_max_ = 0.001
249 parameters	Δρ_max_ = 0.43 e Å^−^^3^
0 restraints	Δρ_min_ = −0.33 e Å^−^^3^

### Special details

**Table:**

Geometry. All esds (except the esd in the dihedral angle between two l.s. planes) are estimated using the full covariance matrix. The cell esds are taken into account individually in the estimation of esds in distances, angles and torsion angles; correlations between esds in cell parameters are only used when they are defined by crystal symmetry. An approximate (isotropic) treatment of cell esds is used for estimating esds involving l.s. planes.
Refinement. Refinement of F^2^ against ALL reflections. The weighted R-factor wR and goodness of fit S are based on F^2^, conventional R-factors R are based on F, with F set to zero for negative F^2^. The threshold expression of F^2^ > 2sigma(F^2^) is used only for calculating R-factors(gt) etc. and is not relevant to the choice of reflections for refinement. R-factors based on F^2^ are statistically about twice as large as those based on F, and R- factors based on ALL data will be even larger.

### Fractional atomic coordinates and isotropic or equivalent isotropic displacement parameters (Å^2^)

**Table:**

	*x*	*y*	*z*	*U*_iso_*/*U*_eq_	
Cl1	0.68839 (13)	0.60065 (10)	0.98954 (7)	0.0933 (4)	
Cl2	0.60921 (11)	0.36582 (8)	0.66235 (7)	0.0709 (3)	
S1	1.08176 (11)	0.90864 (8)	0.17205 (6)	0.0681 (3)	
C1	0.6041 (4)	0.7582 (3)	0.9073 (2)	0.0571 (8)	
C2	0.4887 (4)	0.8210 (4)	0.9701 (3)	0.0747 (10)	
H2	0.4601	0.7782	1.0543	0.090*	
C3	0.4181 (4)	0.9457 (4)	0.9068 (3)	0.0775 (11)	
H3	0.3413	0.9870	0.9483	0.093*	
C4	0.4602 (4)	1.0087 (3)	0.7840 (3)	0.0684 (9)	
H4	0.4126	1.0933	0.7415	0.082*	
C5	0.5738 (3)	0.9472 (3)	0.7221 (2)	0.0510 (7)	
H5	0.6018	0.9919	0.6380	0.061*	
C6	0.6471 (3)	0.8217 (3)	0.7812 (2)	0.0414 (6)	
C7	0.7640 (3)	0.7517 (2)	0.7107 (2)	0.0382 (6)	
H7	0.835 (3)	0.694 (3)	0.758 (2)	0.046*	
C8	0.6777 (3)	0.6492 (2)	0.6789 (2)	0.0351 (5)	
H8	0.645 (3)	0.574 (2)	0.755 (2)	0.042*	
C9	0.5292 (3)	0.7157 (3)	0.6256 (2)	0.0441 (6)	
C10	0.2503 (4)	0.7571 (4)	0.6852 (3)	0.0809 (11)	
H10A	0.1955	0.7190	0.6417	0.097*	
H10B	0.2709	0.8541	0.6349	0.097*	
C11	0.1517 (4)	0.7428 (4)	0.8024 (3)	0.0821 (11)	
H11A	0.1188	0.6484	0.8461	0.123*	
H11B	0.0582	0.8025	0.7881	0.123*	
H11C	0.2133	0.7680	0.8496	0.123*	
C12	0.7963 (3)	0.5897 (2)	0.5979 (2)	0.0369 (6)	
C13	0.7175 (3)	0.5062 (3)	0.5449 (2)	0.0461 (6)	
H13A	0.7998	0.4709	0.4964	0.055*	
H13B	0.6449	0.5662	0.4916	0.055*	
C14	0.8996 (3)	0.8292 (2)	0.5003 (2)	0.0395 (6)	
C15	0.9936 (3)	0.8369 (3)	0.3248 (2)	0.0441 (6)	
C16	1.0255 (5)	0.7895 (3)	0.1151 (3)	0.0830 (11)	
H16A	1.0832	0.7033	0.1477	0.125*	
H16B	1.0510	0.8271	0.0276	0.125*	
H16C	0.9119	0.7740	0.1392	0.125*	
N1	0.8465 (3)	0.8568 (2)	0.59986 (19)	0.0461 (6)	
H1	0.894 (3)	0.929 (3)	0.608 (2)	0.055*	
N2	0.8708 (3)	0.7083 (2)	0.49519 (17)	0.0410 (5)	
N3	0.9342 (3)	0.7110 (2)	0.37947 (17)	0.0417 (5)	
N4	0.9794 (3)	0.9135 (2)	0.39433 (17)	0.0441 (5)	
O1	0.5267 (3)	0.7939 (2)	0.52207 (17)	0.0682 (6)	
O2	0.4005 (2)	0.6814 (2)	0.71234 (18)	0.0611 (6)	
O3	0.9120 (2)	0.51348 (18)	0.66938 (16)	0.0448 (5)	
H3A	0.953 (4)	0.458 (3)	0.647 (3)	0.067*	

### Atomic displacement parameters (Å^2^)

**Table:**

	*U*^11^	*U*^22^	*U*^33^	*U*^12^	*U*^13^	*U*^23^
Cl1	0.1212 (8)	0.0852 (7)	0.0451 (5)	−0.0024 (6)	−0.0173 (5)	0.0064 (4)
Cl2	0.0932 (6)	0.0500 (5)	0.0662 (5)	−0.0327 (4)	−0.0159 (4)	−0.0111 (4)
S1	0.1055 (7)	0.0475 (5)	0.0361 (4)	−0.0024 (4)	0.0124 (4)	−0.0101 (3)
C1	0.073 (2)	0.0615 (19)	0.0356 (14)	−0.0172 (16)	0.0008 (14)	−0.0183 (13)
C2	0.084 (2)	0.101 (3)	0.0403 (16)	−0.028 (2)	0.0177 (16)	−0.0353 (18)
C3	0.078 (2)	0.087 (3)	0.081 (2)	−0.019 (2)	0.020 (2)	−0.056 (2)
C4	0.068 (2)	0.0568 (19)	0.084 (2)	−0.0057 (16)	0.0090 (18)	−0.0391 (17)
C5	0.0643 (18)	0.0407 (16)	0.0465 (15)	−0.0049 (14)	0.0028 (13)	−0.0194 (12)
C6	0.0477 (15)	0.0436 (15)	0.0343 (13)	−0.0110 (12)	0.0024 (11)	−0.0184 (11)
C7	0.0433 (15)	0.0344 (13)	0.0332 (12)	−0.0019 (11)	−0.0027 (11)	−0.0104 (10)
C8	0.0390 (14)	0.0291 (12)	0.0315 (12)	−0.0017 (10)	−0.0041 (10)	−0.0063 (10)
C9	0.0513 (16)	0.0398 (15)	0.0434 (15)	0.0048 (12)	−0.0119 (13)	−0.0179 (12)
C10	0.052 (2)	0.107 (3)	0.089 (3)	0.030 (2)	−0.0287 (18)	−0.043 (2)
C11	0.051 (2)	0.096 (3)	0.109 (3)	0.0175 (19)	−0.021 (2)	−0.051 (2)
C12	0.0450 (14)	0.0256 (12)	0.0385 (13)	−0.0016 (10)	−0.0073 (11)	−0.0102 (10)
C13	0.0582 (17)	0.0355 (14)	0.0434 (14)	−0.0058 (12)	−0.0109 (12)	−0.0117 (11)
C14	0.0477 (15)	0.0316 (13)	0.0369 (13)	−0.0033 (11)	0.0007 (11)	−0.0136 (10)
C15	0.0556 (16)	0.0331 (14)	0.0355 (13)	0.0054 (12)	0.0031 (11)	−0.0107 (11)
C16	0.141 (3)	0.067 (2)	0.0378 (16)	0.008 (2)	−0.0113 (18)	−0.0201 (15)
N1	0.0583 (14)	0.0386 (12)	0.0421 (12)	−0.0177 (11)	0.0107 (10)	−0.0208 (10)
N2	0.0537 (13)	0.0327 (11)	0.0351 (11)	−0.0054 (10)	0.0035 (9)	−0.0150 (8)
N3	0.0580 (14)	0.0316 (11)	0.0339 (11)	0.0016 (10)	−0.0019 (9)	−0.0138 (9)
N4	0.0567 (14)	0.0328 (11)	0.0374 (11)	−0.0029 (10)	0.0074 (10)	−0.0137 (9)
O1	0.0783 (15)	0.0711 (14)	0.0463 (12)	0.0242 (12)	−0.0219 (10)	−0.0138 (10)
O2	0.0390 (11)	0.0711 (14)	0.0615 (12)	0.0076 (10)	−0.0113 (9)	−0.0140 (10)
O3	0.0513 (11)	0.0359 (10)	0.0513 (11)	0.0109 (8)	−0.0149 (9)	−0.0207 (8)

### Geometric parameters (Å, °)

**Table:**

Cl1—C1	1.725 (3)	C10—O2	1.454 (3)
Cl2—C13	1.773 (2)	C10—C11	1.465 (5)
S1—C15	1.740 (2)	C10—H10A	0.9700
S1—C16	1.783 (4)	C10—H10B	0.9700
C1—C6	1.392 (3)	C11—H11A	0.9600
C1—C2	1.404 (4)	C11—H11B	0.9600
C2—C3	1.374 (5)	C11—H11C	0.9600
C2—H2	0.9300	C12—O3	1.401 (3)
C3—C4	1.356 (4)	C12—N2	1.456 (3)
C3—H3	0.9300	C12—C13	1.526 (3)
C4—C5	1.381 (4)	C13—H13A	0.9700
C4—H4	0.9300	C13—H13B	0.9700
C5—C6	1.378 (4)	C14—N4	1.332 (3)
C5—H5	0.9300	C14—N1	1.337 (3)
C6—C7	1.517 (4)	C14—N2	1.342 (3)
C7—N1	1.458 (3)	C15—N3	1.321 (3)
C7—C8	1.554 (3)	C15—N4	1.360 (3)
C7—H7	0.92 (3)	C16—H16A	0.9600
C8—C9	1.502 (3)	C16—H16B	0.9600
C8—C12	1.539 (3)	C16—H16C	0.9600
C8—H8	0.96 (2)	N1—H1	0.93 (3)
C9—O1	1.198 (3)	N2—N3	1.393 (3)
C9—O2	1.329 (3)	O3—H3A	0.77 (3)
			
C15—S1—C16	101.76 (14)	C10—C11—H11A	109.5
C6—C1—C2	120.3 (3)	C10—C11—H11B	109.5
C6—C1—Cl1	121.1 (2)	H11A—C11—H11B	109.5
C2—C1—Cl1	118.6 (2)	C10—C11—H11C	109.5
C3—C2—C1	119.9 (3)	H11A—C11—H11C	109.5
C3—C2—H2	120.0	H11B—C11—H11C	109.5
C1—C2—H2	120.0	O3—C12—N2	109.8 (2)
C4—C3—C2	120.2 (3)	O3—C12—C13	113.3 (2)
C4—C3—H3	119.9	N2—C12—C13	107.02 (19)
C2—C3—H3	119.9	O3—C12—C8	105.82 (18)
C3—C4—C5	120.0 (3)	N2—C12—C8	106.35 (18)
C3—C4—H4	120.0	C13—C12—C8	114.3 (2)
C5—C4—H4	120.0	C12—C13—Cl2	111.07 (17)
C6—C5—C4	122.1 (3)	C12—C13—H13A	109.4
C6—C5—H5	119.0	Cl2—C13—H13A	109.4
C4—C5—H5	119.0	C12—C13—H13B	109.4
C5—C6—C1	117.5 (2)	Cl2—C13—H13B	109.4
C5—C6—C7	121.2 (2)	H13A—C13—H13B	108.0
C1—C6—C7	121.2 (2)	N4—C14—N1	127.2 (2)
N1—C7—C6	109.5 (2)	N4—C14—N2	110.8 (2)
N1—C7—C8	110.5 (2)	N1—C14—N2	121.9 (2)
C6—C7—C8	111.7 (2)	N3—C15—N4	116.1 (2)
N1—C7—H7	112.1 (16)	N3—C15—S1	124.2 (2)
C6—C7—H7	111.0 (16)	N4—C15—S1	119.66 (19)
C8—C7—H7	101.9 (16)	S1—C16—H16A	109.5
C9—C8—C12	113.9 (2)	S1—C16—H16B	109.5
C9—C8—C7	110.8 (2)	H16A—C16—H16B	109.5
C12—C8—C7	110.40 (19)	S1—C16—H16C	109.5
C9—C8—H8	108.1 (15)	H16A—C16—H16C	109.5
C12—C8—H8	107.5 (14)	H16B—C16—H16C	109.5
C7—C8—H8	105.7 (14)	C14—N1—C7	120.7 (2)
O1—C9—O2	124.5 (3)	C14—N1—H1	118.2 (17)
O1—C9—C8	125.5 (3)	C7—N1—H1	117.4 (16)
O2—C9—C8	109.9 (2)	C14—N2—N3	109.23 (18)
O2—C10—C11	107.0 (3)	C14—N2—C12	124.9 (2)
O2—C10—H10A	110.3	N3—N2—C12	125.74 (19)
C11—C10—H10A	110.3	C15—N3—N2	101.43 (19)
O2—C10—H10B	110.3	C14—N4—C15	102.3 (2)
C11—C10—H10B	110.3	C9—O2—C10	118.2 (2)
H10A—C10—H10B	108.6	C12—O3—H3A	111 (2)
			
C6—C1—C2—C3	−0.1 (5)	N2—C12—C13—Cl2	−175.47 (17)
Cl1—C1—C2—C3	−179.3 (3)	C8—C12—C13—Cl2	−58.0 (2)
C1—C2—C3—C4	−0.4 (5)	C16—S1—C15—N3	12.5 (3)
C2—C3—C4—C5	0.3 (5)	C16—S1—C15—N4	−165.8 (2)
C3—C4—C5—C6	0.3 (5)	N4—C14—N1—C7	−178.5 (2)
C4—C5—C6—C1	−0.7 (4)	N2—C14—N1—C7	4.3 (4)
C4—C5—C6—C7	176.2 (3)	C6—C7—N1—C14	−151.3 (2)
C2—C1—C6—C5	0.6 (4)	C8—C7—N1—C14	−27.9 (3)
Cl1—C1—C6—C5	179.8 (2)	N4—C14—N2—N3	−0.1 (3)
C2—C1—C6—C7	−176.3 (3)	N1—C14—N2—N3	177.5 (2)
Cl1—C1—C6—C7	2.8 (4)	N4—C14—N2—C12	175.4 (2)
C5—C6—C7—N1	30.3 (3)	N1—C14—N2—C12	−7.0 (4)
C1—C6—C7—N1	−152.8 (2)	O3—C12—N2—C14	−82.2 (3)
C5—C6—C7—C8	−92.4 (3)	C13—C12—N2—C14	154.4 (2)
C1—C6—C7—C8	84.5 (3)	C8—C12—N2—C14	31.9 (3)
N1—C7—C8—C9	−74.3 (3)	O3—C12—N2—N3	92.6 (3)
C6—C7—C8—C9	47.9 (3)	C13—C12—N2—N3	−30.8 (3)
N1—C7—C8—C12	52.8 (3)	C8—C12—N2—N3	−153.3 (2)
C6—C7—C8—C12	175.02 (19)	N4—C15—N3—N2	1.9 (3)
C12—C8—C9—O1	−45.3 (3)	S1—C15—N3—N2	−176.39 (19)
C7—C8—C9—O1	79.9 (3)	C14—N2—N3—C15	−1.1 (3)
C12—C8—C9—O2	138.4 (2)	C12—N2—N3—C15	−176.5 (2)
C7—C8—C9—O2	−96.4 (2)	N1—C14—N4—C15	−176.3 (3)
C9—C8—C12—O3	−170.66 (19)	N2—C14—N4—C15	1.2 (3)
C7—C8—C12—O3	64.0 (2)	N3—C15—N4—C14	−2.0 (3)
C9—C8—C12—N2	72.6 (2)	S1—C15—N4—C14	176.38 (19)
C7—C8—C12—N2	−52.8 (2)	O1—C9—O2—C10	−8.7 (4)
C9—C8—C12—C13	−45.3 (3)	C8—C9—O2—C10	167.6 (3)
C7—C8—C12—C13	−170.64 (19)	C11—C10—O2—C9	−157.3 (3)
O3—C12—C13—Cl2	63.3 (3)		

### Hydrogen-bond geometry (Å, °)

**Table:**

*D*—H···*A*	*D*—H	H···*A*	*D*···*A*	*D*—H···*A*
N1—H1···N4^i^	0.93 (3)	2.04 (3)	2.969 (3)	172 (2)
O3—H3A···N3^ii^	0.77 (3)	2.05 (3)	2.806 (3)	170 (3)
C16—H16A···O3^ii^	0.96	2.47	3.290 (4)	143

Symmetry codes: (i) −*x*+2, −*y*+2, −*z*+1; (ii) −*x*+2, −*y*+1, −*z*+1.
